# Telerehabilitation: Exploring the Untapped Potential

**DOI:** 10.7759/cureus.57405

**Published:** 2024-04-01

**Authors:** Saurabh Agnihotri, Nalina Gupta, Pooja Sindwani, Ankita Srivastava, Aftab Ahmad, Medha Karki

**Affiliations:** 1 Physiotherapy, Teerthanker Mahaveer University, Moradabad, IND; 2 Neurological Physiotherapy and Community Rehabilitation, College of Physiotherapy, Sumandeep Vidyapeeth Deemed to be University, Vadodara, IND; 3 Community Medicine, Teerthanker Mahaveer Medical College and Research Centre, Moradabad, IND

**Keywords:** neuro telerehabilitation, cardiac telerehabilitation, virtual rehabilitation, telephysiotherapy, telerehabilitation

## Abstract

Telerehabilitation is a burgeoning field that holds immense promise in revolutionizing the delivery of rehabilitation services. Defined as a branch of telecommunication utilizing technologies such as the internet, it facilitates remote interaction between healthcare providers and patients, transcending geographical barriers. This method proves invaluable in patient assessment, counseling, and treatment across various medical domains, including physical therapy, speech therapy, psychotherapy, and occupational therapy. Particularly beneficial for individuals with disabilities or those unable to access traditional healthcare facilities, telerehabilitation mitigates the constraints of time and cost associated with travel. This paper explores the evolution, types, uses, and research findings in telerehabilitation, shedding light on its transformative potential in health care.

## Editorial

Telecommunication-based practices in physiotherapy are one of the newer evolutions in the field of rehabilitation and are popularly known as "Telerehabilitation." Telerehabilitation is defined as the use of telecommunication technologies to provide distance therapeutic rehabilitation. It comprises interventions like exercises, education, and pain control in the absence of a healthcare specialist. This newer advancement in the field of rehabilitation is primarily designed for patients who have been transferred back to their homes after the prescribed period of hospitalization. Patient-rehabilitator interaction is the substitute for the traditional face-to-face approach of the older version of rehabilitation [1]. Musculoskeletal conditions, dermatological disorders, stroke, oral diseases, and pulmonary disorders are some of the fields wherein monitoring and providing appropriate healthcare and rehabilitation services are done via various telecommunication technologies included in telerehabilitation. Nowadays, telerehabilitation is also used for remote consultations, as a modality to provide easily accessible service. Recently, home-based services have also been substituted by tele-homecare programs [2]. Telerehabilitation via gaming techniques like robotics, virtual reality as well as augmented reality (AR) have been proven effective in the rehabilitation of stroke [3]. Through telerehabilitation techniques, monitoring of cardiovascular parameters like ECG and blood pressure has also been done using Android tablets and the Internet of Things (IoT) in cardiac rehabilitation programs [4].

Evolution of telerehabilitation

Telerehabilitation first came into the limelight in 1999. Since the 1950s, D.M. Angaran focused on catering an encyclopedic history of telemedicine, telecommunication, and the internet. Telemedicine was also used by the National Aeronautics and Space Administration (NASA) during various space programs. The Department of Defense (DOD) used telerehabilitation during the Vietnam War. Rural healthcare systems, the radiology profession, and state penitentiary systems were the lighthouse customers of telemedicine. In 2002, the State of Science Conference assembled a group of fascinated government officials, engineers, and clinicians (both civil and military) to use telecommunication for rehabilitation therapy and assessment. The first internet software for telerehabilitation was made by O. Bracy, a neuropsychologist, in 2001 on cognitive rehabilitation therapy. In 2006, the article "Telemedicine as a Profitable Business for Hospitals" was published by M.J. McCue and S.E. Palsbo [5].

Types of telerehabilitation

Telerehabilitation uses a variety of technologies that aid in guiding the patient to perform accurate exercises prescribed by his/her physical therapist. Plain old telephone service, videotelephony, virtual reality, web-based approaches, sensor and body monitoring, wireless technology, mobile telephony, mobile applications, interactive virtual therapy (IVT), virtual reality balance training (VRBT), and robotics are some of the technologies that are being used worldwide in rehabilitation. Plain old telephone service is the oldest technology used in telerehabilitation, which focuses on bi-directional communication. Videotelephony is a mode of telerehabilitation that uses videoconferencing and webcams to assist the treatment. Video-based telerehabilitation is effective in patients with knee osteoarthritis [2]. Studies have shown that videoconferencing led to significant improvement in outcome measures in patients with chronic obstructive pulmonary disorder [6]. Virtual reality is a computer-based technology that allows rehabilitation in the virtual world via video games, thus enhancing auditory and visual feedback. IVT and VRBT are the common types of virtual reality technologies used in telerehabilitation. VRBT has proved to help improve balance and posture in stroke patients [4,7]. IVT is proven to be equally effective as conventional physical therapy in post-total knee arthroplasty (TKA) patients [8]. The advancements made in the field of AR technology also hold promising avenues for telerehabilitation. Web-based approaches like software or applications are used by both the therapist and patient to assist in rehabilitation. Adaptive cognitive remediation (ACR) program, a web-based approach, has been proven to help improve the cognitive functions in multiple sclerosis (MS) and other neurological conditions in combination with cognitive dysfunctions [9]. Telerehabilitation via messages, email, and videos is also helpful in treating and rehabilitating the condition of the patient. Telerehabilitation through smartphone apps via secure chat and video calls has shown a marked decrease in pain, disability rate, analgesic consumption, mental health, and surgery intent in patients with acute low back pain (LBP) [10]. Sensors and wearable devices are another advancement in telerehabilitation. Wireless sensors like accelerometers, gyroscopes, and inertial motion units (IMUs) are the commonly used sensors for monitoring body movements, like angulation and velocity, during the prescribed treatment. Telerehabilitation via IMU-based sensors showed marked reductions in disability, pain, mental health, analgesic usage, and surgery intent in patients with wrist pain [11]. Telerehabilitation via wearable inertial measurement units (IMU-based sensors) consisting of a gyroscope and accelerometer proved to be significantly effective in improving shoulder range of motion (ROM) in patients with adhesive capsulitis [12]. These newly evolved technologies help healthcare professionals to monitor the health and development of the patient. These aforementioned varieties of telerehabilitation are also useful in monitoring, intervention, supervision, education, assessment, consultation, counseling, and prevention.

Uses of telerehabilitation

Telerehabilitation is useful as it overcomes the barrier of distance of communication, thus enabling and enhancing a more cost-effective physical rehabilitation. Telerehabilitation has resulted in a decrease in the number of outpatient visits, thus making it easier for patients to continue the prescribed exercises most appropriately. This is an additional advantage in developing countries like India, where a significant proportion of the population are daily wage earners. VRBT improves balance and posture along with a significant reduction in several outpatient visits in patients with stroke, thus, reducing the cost-related issues faced by patients during their respective rehabilitation [7]. Telerehabilitation also plays a vital role in the reduction in the length of stay in hospitals. It has been established that pre-rehabilitation via telerehabilitation is useful in reducing the length of stay in the hospital, and significant improvement in the condition of the patient at the time of discharge when compared to patients who underwent standard preoperative protocol for TKA [13]. Telerehabilitation is also useful in decreasing the gap in cases where patients did not get the best guidance to return for sports rehabilitation. Telerehabilitation may also be beneficial for patients who undergo anterior cruciate ligament reconstruction (ACLR) [14].

Research on telerehabilitation and physiotherapy

Telerehabilitation is still in its nascent stages and its utility is still being explored. Nowadays, telerehabilitation plays a vital role in the field of physiotherapy. Telerehabilitation is the newer advancement in the field of physical therapy wherein the patient and therapist interact with one another, without physically meeting regularly. Telerehabilitation focuses on improving health outcomes for various fields of physical therapy like orthopedics, neurology, cardiology, and pulmonary medicine.

Telephysiotherapy and musculoskeletal conditions

Musculoskeletal pain is associated with impaired productivity and high economic burden. Telerehabilitation focuses on imparting therapeutic impacts like reduction in pain and improvement in physical function. It also aims at providing an effective substitute in cases wherein the patient is unable to visit the healthcare professional due to pain. In such a scenario, telerehabilitation has proven to be a boon to regain muscular strength through exercises prescribed by the therapist via the use of the internet. In musculoskeletal conditions, telerehabilitation primarily focuses on improving health outcomes for musculoskeletal disorders like wrist pain, knee osteoarthritis, adhesive capsulitis, LBP, shoulder pain, and sprain. The effectiveness of telerehabilitation in various musculoskeletal pain is supported by some evidence-based research. Telerehabilitation via videoconferencing is superior to home-based exercise programs, including telephonic consultation in patients with knee osteoarthritis [15]. Telerehabilitation has been established to provide positive therapeutic results for pain management and functions in patients with musculoskeletal conditions [16]. Telerehabilitation is feasible in attaining clinical outcomes like improvement in activities of daily living and pain compared to face-to-face treatment in patients with ankle sprain [17]. Telerehabilitation through motion sensors is useful in improving the ROM in adhesive capsulitis [12]. Telerehabilitation is also a promising and viable option in the holistic management post musculoskeletal surgeries. Telerehabilitation boosts more effective and successful improvement in overall physical functions in post-TKA patients [1]. Telerehabilitation is more effective in improving outcome scores than conventional therapy for shoulder rehabilitation post arthroscopic rotator cuff repair (ARCR) [18]. Telerehabilitation helps to decrease the gap for athletic patients who did not get the best guidance to return for sports rehabilitation in post-ACLR surgery cases [14].

Telephysiotherapy and neurological conditions

Telerehabilitation has also proved to be a viable option in the holistic treatment and management of various neurological disorders and pain. Telerehabilitation is effective in treating stroke patients, along with its cost-effectiveness [19]. Cognitive training through telerehabilitation can be used in the rehabilitation of patients with learning disabilities [20]. ACR programs can improve cognitive functions in MS cases [9]. VRBT (virtual reality-supported balanced approach of telerehabilitation) improves balance and postural functions in patients with stroke with balance deficits and helps in the reduction of outpatient visits and cost-related issues [7]. It not only focuses on educating the patient but also focuses on educating the caregivers to provide the required rehabilitative exercises (caregiver-mediated exercises) to the patient [19].

Telephysiotherapy and cardio-pulmonary conditions

Telerehabilitation also seems to help provide holistic treatment and management in the cardiopulmonary field. During COVID-19, the improvement in the functional recovery of the patients was significant and clinically more in the telerehabilitation group than the control group [21]. The home-based cardiac telerehabilitation increases cardio-respiratory fitness in patients suffering from coronary heart disease [22]. Telerehabilitation can improve the quality of life of heart failure patients by increasing mental, social, and sexual functions along with an increase in exercise capacity and reduction of symptoms, thus helping in regaining independence in carrying out activities of daily living [23].

Telephysiotherapy and pediatric conditions

Telerehabilitation has extended its roots and proved effective in pediatric cases. Researches show that it can be considered an easy approachable rehabilitation service for children who require physiotherapy treatment for any prevailing health conditions living in geographically remote areas during the COVID-19 pandemic [24].

Conclusion

Telerehabilitation has shown a positive impact in many medical fields. It has also proved to be as effective as conventional face-to-face rehabilitation programs. It is a progressing field that has the opportunity to foster fascinating rehabilitation paradigms with an appreciable cost-benefit ratio. It can be used in emergency conditions like sudden occurrence of the disease wherein contacting the healthcare professional can help the patient to recover from the disease and thus impede the severity of the disease. Further research studies are warranted in the field of telerehabilitation to tap into its full potential.
